# Antibacterial and Biodegradable Polysaccharide-Based Films for Food Packaging Applications: Comparative Study

**DOI:** 10.3390/ma15093236

**Published:** 2022-04-29

**Authors:** Weronika Janik, Michał Nowotarski, Divine Yutefar Shyntum, Angelika Banaś, Katarzyna Krukiewicz, Stanisław Kudła, Gabriela Dudek

**Affiliations:** 1 Łukasiewicz Research Network—The Institute of Heavy Organic Synthesis “Blachownia”, Energetyków 9, 47-225 Kędzierzyn-Koźle, Poland; stanislaw.kudla@icso.lukasiewicz.gov.pl; 2 Department of Physical Chemistry and Technology of Polymers, PhD School, Silesian University of Technology, 2a Akademicka Str., 44-100 Gliwice, Poland; 3 Department of Physical Chemistry and Technology of Polymers, Faculty of Chemistry, Silesian University of Technology, Strzody 9, 44-100 Gliwice, Poland; michnow566@student.polsl.pl (M.N.); angeban429@student.polsl.pl (A.B.); katarzyna.krukiewicz@polsl.pl (K.K.); gabriela.maria.dudek@polsl.pl (G.D.); 4 Biotechnology Centre, Silesian University of Technology, B. Krzywoustego 8, 44-100 Gliwice, Poland; divine.yufetar.shyntum@polsl.pl

**Keywords:** starch, chitosan, alginate, biopolymers, polysaccharide, modification, barrier properties

## Abstract

One of the major objectives of food industry is to develop low-cost biodegradable food packaging films with optimal physicochemical properties, allowing for their large-scale production and providing a variety of applications. To meet the expectations of food industry, we have fabricated a series of solution-cast films based on common biodegradable polysaccharides (starch, chitosan and alginate) to be used in food packaging applications. Selected biopolymers were modified by the addition of glycerol and oxidized sucrose (starch), glycerol (chitosan), and glycerol and calcium chloride (alginate), as well as being used to form blends (starch/chitosan and starch/alginate, respectively). A chestnut extract was used to provide antibacterial properties to the preformed materials. The results of our studies showed that each modification reduced the hydrophilic nature of the polymers, making them more suitable for food packaging applications. In addition, all films exhibited much higher barrier properties to oxygen and carbon dioxide than commercially available films, such as polylactic acid, as well as exhibiting antimicrobial properties against model Gram-negative and Gram-positive bacteria (*Escherichia coli* and *Staphylococcus epidermidis,* respectively)*,* as well as yeast (*Candida albicans*).

## 1. Introduction

As the environment becomes increasingly polluted with plastics, there is an urgent need for the development of biodegradable polymers applicable in industry. Currently, these are polysaccharides, such as starch, chitosan and alginates, that serve as the most popular biodegradable polymers. However, the raw polysaccharide-based materials do not exhibit the appropriate physicochemical properties necessary for food packaging applications [1,2,3,4]. Therefore, numerous recent studies [3,5,6,7,8,9] focus on the modification of such biopolymers. Unfortunately, the modification of biodegradable polymers is not as obvious as in the case of conventional synthetic polymers, since each polysaccharide, due to its chemical structure, requires a different modification method. Additionally, it is known that the introduction of some modifications can have an adverse effect on the process of biodegradation. Since biodegradable polymers are primarily used in the packaging market, modifiers must be non-hazardous and considered as safe for food contact. 

The most common modification route for starch is plasticization, and the most commonly used plasticizer is glycerol [5,6,7]. This modification is used to improve the elongation, distribution, flexibility, elasticity, and rigidity of the film [8,9,10,11]. Moreover, the addition of plasticizers can change the continuity and thus improve the properties of starch-based films [5]. Another popular way to modify starch is by using crosslinking, e.g., with natural organic acids (lactic acid, malic acid and citric acid). The presence of acidic crosslinking agents leads to the decrease in swelling, water uptake and water vapor permeability of materials, and these effects are proportional to the amount of a crosslinker [12,13,14]. Furthermore, the presence of organic acids was found to improve the tensile strength of starch films [15,16], as well as to reduce water vapor permeability, and to increase thermal properties without affecting biodegradability of investigated films [17,18]. Xu et al. [19] used oxidized sucrose as a starch crosslinking agent, and the obtained films were found to be strong and ductile. 

Chitosan can be modified through the addition of plasticizers or crosslinking agents (citric acid) [20,21,22,23,24]. Similarly to the case of starch, plasticizers improve the elongation, distribution, flexibility, elasticity, and rigidity of chitosan-based films [25,26,27]. Chitosan, due to its chemical nature, does not always require additional modifications beyond plasticization. It dissolves only in acidic conditions, so it is not as highly soluble in water as other polysaccharides. Nevertheless, crosslinking with citric acid can provide some additional benefits, for instance the improvement in mechanical, thermal and moisture barrier properties when compared to the unmodified chitosan film [21,22]. Another approach for chitosan modification is the use of glycidol [28,29,30,31]. The resulting chitosan derivatives form micelles, hydrogels or membranes, and are commonly used for drug/gene delivery.

For sodium alginates, the most common modification is the use of calcium chloride as a crosslinking agent [32,33,34]. Recent results indicate that the swelling properties of alginate films decrease after the addition of CaCl_2_, which is due to the crosslinking reaction between the carboxyl group of alginate and calcium ions [33,35,36]. Modified alginate films exhibited higher tensile stress and tensile strain compared to the unmodified films [33,34]. Giz et al. [34] revealed a synergistic effect when alginate films were crosslinked with CaCl_2_ and plasticized with glycerol, as they observed an increase in tensile strength and a decrease in elongation at break as the result of this “double” modification route.

Another approach to achieve suitable physical and mechanical properties of biodegradable films is to fabricate blends by mixing two or three polymers. The results of infrared spectroscopy, scanning electron microscopy and X-ray diffraction have confirmed the compatibility between starch, chitosan and alginate, due to the presence of strong interactions such as hydrogen bonds, as well as ionic interactions [37,38,39]. Moreover, such films are generally homogeneous, thin, smooth, with good coherence and no visual defects [40,41]. 

Numerous comparisons of the same non-modified and modified biodegradable polymers can be found in the literature [32,42,43,44,45,46]. Unfortunately, there is still limited data showing how polymers of different chemical structures belonging to the same group differ in their properties. Therefore, we have decided to perform a comparative study using three non-modified and modified polysaccharides, namely starch, chitosan and alginate. In the present study, starch was modified by the addition of oxidized sucrose (crosslinking agent) and glycerol (plasticizer), chitosan was modified by the addition of glycerol (plasticizer), and alginate was modified by the addition of CaCl_2_ (crosslinking agent) and glycerol (plasticizer). Additionally, we have fabricated polymer blends by mixing two polysaccharides to form starch/chitosan and starch/alginate, respectively. All materials were modified with a chestnut extract, which is known from its antimicrobial activity [47]. Preformed films were characterized to assess their applicability as food packaging materials, and the following properties were determined: hydrophilic properties (moisture content, swelling degree, total soluble matter, water contact angle), barrier properties (oxygen and carbon dioxide permeability), and mechanical properties (tensile strength, elongation at break). Furthermore, antimicrobial properties of developed films, which are of great interest for storing food, were also investigated. The performance of polysaccharide-based films was compared with the performance of a commercially available poly(lactic acid) (PLA) film. 

## 2. Materials and Methods

### 2.1. Materials

Starch was purchased from Heuschen & Schrouff OFT B.V. Chitosan 30–100 cps (MW = 250,000, DD ≥ 90%) was purchased from Sigma-Aldrich (St. Louis, MI, USA). Sodium alginate (Brookfield viscosity 350–550 mPas, c = 1 wt.% at 20 °C) was supplied by Acros Organics (Branchburg, NJ, USA). Acetic acid was purchased from POCH S. A. (Gliwice, Poland) (99.5–99.9%) and chestnut extract Farmatan (≥76% tannins) was provided by Tanin Sevnica (Sevnica, Slovenia). Calcium chloride (purity ≥ 96%) was purchased from Avantor Performance Materials (Radnor, PA, USA). Glycerol was produced by Nortchem (Los Angeles, CA, USA). Oxidized sucrose was prepared using sucrose from Pfeifer & Langen Polska (Poznań, Poland), sodium periodate (>98%) from Acros Organics and barium chloride purchased from STANLAB Sp. J. Lublin (Lublin, Poland). 

### 2.2. Film Preparation

#### 2.2.1. Modified Starch-Based Films

##### Preparation of Oxidized Sucrose

Oxidized sucrose was prepared according to a slightly modified protocol described in Wang et al. [48]. In short, 6.50 g sucrose and 12.90 g sodium periodate were dissolved in 200 mL distilled water and stirred at room temperature (24 °C) on a magnetic stirrer (IKA, Staufen, Germany) at 1000 rpm for 24 h. Approximately 7 g of barium chloride was then added to the solution and stirred at 5 °C for 1 h to allow complete precipitation. The solution was filtered, and the filtrate was stored in a fridge (5 °C) for further use.

##### Preparation of Modified Starch-Based Films

Starch solution (5%, *w*/*w*) was prepared by dispersing starch with chestnut extract (0.75% *w*/*v*) and glycerol (40%, *w*/*w* based on the mass of starch) in water. Oxidized sucrose (15 % *w*/*w* based on the mass of starch) was added, and the mixture was stirred at 90 °C for 30 min. The solution was poured onto Teflon-coated plates. The cast films were dried overnight at room temperature (24 °C) and later peeled from the plates. The starch films were then incubated/dried in an air oven at 160 °C for 4 min.

#### 2.2.2. Modified Chitosan-Based Films

Chitosan solution (2%, *w*/*w*) was prepared by dispersing chitosan in acetic acid solution (1%, *v*/*v*) with chestnut extract (0.75% *w*/*v*) and glycerol (30%, *w*/*w* based on the mass of chitosan). The mixture was agitated overnight on a magnetic stirrer (IKA, Staufen, Germany) at 1000 rpm and room temperature (24 °C). Afterwards, the mixture was homogenized at 6000 rpm for 5 min with an homogenizator (Ultra-Turrax T50, IKA, Staufen, Germany) and left overnight. The mixture was cast on Petri dishes and left overnight at room temperature (24 °C) to dry.

#### 2.2.3. Modified Alginate-Based Films

Alginate solution (1%, *w*/*w*) was prepared by dispersing sodium alginate with chestnut extract (0.75% *w/v*) and glycerol (30%, *w*/*w* based on the mass of alginate) in water and stirring overnight on a magnetic stirrer (IKA, Staufen, Germany) at 1000 rpm and room temperature (24 °C). Afterwards, the mixture was homogenized at 6000 rpm for 5 min with an homogenizator (Ultra-Turrax T50, IKA, Staufen, Germany) and left overnight. The mixture was cast on Petri dishes and left overnight at room temperature (24 °C) to dry. The cast films were peeled from the plates and soaked in 40 mL 2.5% CaCl_2_ solution for 2 h. Thereafter, the films were washed with distilled water and dried at room temperature.

#### 2.2.4. Modified Starch and Chitosan-Based Films

Starch and chitosan solution was prepared by dispersing starch (2%, *w*/*w*) and chitosan (2%, *w*/*w*) with chestnut extract (0.75% *w*/*v*) and glycerol (30%, *w*/*w* based on the mass of starch and chitosan) in acetic acid solution (1%, *v*/*v*), and stirring overnight on a magnetic stirrer (IKA, Staufen, Germany) at 1000 rpm and at 100 °C. Afterwards, the mixture was homogenized for 5 min at 6000 rpm with an homogenizator (Ultra-Turrax T50, IKA, Staufen, Germany) and left overnight. Thereafter, oxidized sucrose (15% *w*/*w* based on the mass of starch) was added and stirred at 90 °C for 30 min. The solution was poured onto Petri dishes and the cast films were dried overnight at room temperature (24 °C) and later peeled from the plates. The films were then treated in an air oven at 160 °C for 4 min. 

#### 2.2.5. Modified Starch and Alginate-Based Films

Starch and alginate solution was prepared by dispersing starch (5%, *w*/*w*) and alginate (1%, *w*/*w*) with chestnut extract (0.75% *w*/*v*) and glycerol (30%, *w*/*w* based on the mass of starch and alginate) in distilled water and stirring overnight on a magnetic stirrer at 1000 rpm and at 100 °C. Afterwards, the mixture was homogenized at 6000 rpm for 5 min with an homogenizator (Ultra-Turrax T50, IKA, Staufen, Germany) and left overnight. Then, oxidized sucrose (10% *w*/*w* based on the mass of starch) was added and stirred at 90 °C for 30 min. The solution was poured onto Petri dishes and the cast films were dried overnight at room temperature (24 °C) and later peeled from the plates. The films were then treated in an air oven at 160 °C for 4 min. The abbreviations of all obtained films are shown in Table 1. 

### 2.3. Measurement of Film Thickness

The film thickness was measured with a digimatic micrometer (Mitutoyo Absolute, Tester Sangyo Co., Ltd., Tokyo, Japan). Twenty values were randomly taken at different locations for each film, and the average value was determined as the final result. 

### 2.4. Measurement of Moisture Content, Swelling Degree and Total Soluble Matter

Moisture content (*MC*), swelling degree (*SD*) and total soluble matter (*TSM*) were determined using a gravimetric three-step method. Square samples with an area of 1 cm^2^ were cut from the films and weighed on an analytical balance (*M_1_*). Then, the samples were dried at 100 °C for 24 h and weighed again (*M_2_*). Thereafter, the analyzed film pieces were placed in 30 mL of distilled water, left at room temperature (24 °C) for 24 h and weighed again (*M_3_*). In the last step, the samples were dried at 100 °C for 24 h and weighed (*M_4_*). The measurements were repeated five times and the average value was determined. *MC*, *SD* and *TSM* were calculated as follows:

(1) $$MC\left(\%\right)=\frac{\left(M_{1}-M_{2}\right)}{M_{1}}\times 100$$

(2) $$SD\left(\%\right)=\frac{\left(M_{3}-M_{2}\right)}{M_{2}}\times 100$$

(3) $$TSM\left(\%\right)=\frac{\left(M_{2}-M_{4}\right)}{M_{2}}\times 100$$

### 2.5. Measurement of Contact Angle

The contact angle between water and the films was measured using an optical contact angle meter and a contour analysis systems (OCA15 from DataPhysic, Filderstadt, Germany) at room temperature (24 °C). Deionized water (1 μL) was carefully dropped onto the films and contact angles were determined from the average of ten measurements. 

### 2.6. Measurement of Oxygen and Carbon Dioxide Permeability

Oxygen and carbon dioxide permeability was determined using an isobaric apparatus (Supplementary Information, Figure S1). The samples in a form of a disk with an area of about 6 cm^2^ were sealed in a diffusion chamber. In the chamber, compressed oxygen (class 5.0) or carbon dioxide (technical gas) were supplied at a controlled flow rate to keep the pressure constant in that compartment. The flow of the chambers was connected to a manometer to ensure the equality of pressures (from 0.05 to 1.0 MPa). 

Before the measurement, the samples were degassed for 24 h and then placed in the apparatus to condition for 2 h with the appropriate gas. The permeation coefficient was determined as follows:

(4) $$P=\frac{V\times l}{S\times \Delta p}$$

where *V* is the volumetric flow (mol∙s^−1^), *l* is the sample thickness (m), *S* is the sample activate area (m^2^) and Δ*p* is the pressure difference on both sides of the film (Pa).

### 2.7. Measurement of Mechanical Properties

Mechanical properties, including tensile strength and elongation at break, were performed by means of an Instron 4466 machine at a speed of 5 mm/min at room temperature. Films were cut into strips (80 mm × 20 mm) for measurement. Final values of tensile strength and elongation at break were determined from the average of ten measurements.

### 2.8. Measurement of Antimicrobial Properties

Before using the chestnut extract for the fabrication of polysaccharide-based foils, its antimicrobial properties were assessed against model Gram-positive bacteria *(Staphylococcus epidermidis* ATCC12228), Gram-negative bacteria (*Escherichia coli* ATCC25922) and yeasts (*Candida albicans* ATCC18804) using an inhibition zone assay [49] (Supplementary Information, Figure S2). This experiment confirmed superior antimicrobial properties of chestnut extract, particularly when compared with other investigated plant extracts, including those derived from nettle, grape, and graviola.

Antimicrobial activity of polysaccharide films was determined against standard bacterial isolates, i.e., Gram-negative bacteria *Escherichia coli* ATCC25922 (*E. coli*) and Gram-positive bacteria *Staphylococcus epidermidis* ATCC12228 (*S. epidermidis*), as well as yeast (*Candida albicans* ATCC18804), by a serial dilution following co-culture with each film. Briefly, a single bacterial colony was cultured in 10 mL of Mueller-Hinton broth (MHB) at 37 °C overnight. Thereafter, bacteria from a 1 mL of cultures were collected by centrifugation (7000× *g*, 3 min, 4 °C), and resuspended in a sterile saline solution. The optical density was normalized to 0.5 McFarland standard (approximately 1.5 × 10^8^ CFU/mL) using a densitometer. Finally, 100 µL of the normalized cultures were inoculated into sterile bottles containing 4 mL M9 minimal supplemented glucose and the given film to be analyzed. The resulting mixture was then incubated overnight at 37 °C in a shaking incubator, followed by a serial dilution plating on MHB for CFU/mL determination. Experiments were repeated four times and the results presented as the log_10_ of the average CFU/mL.

### 2.9. Scanning Electron Microscopy

Surface morphology of the films was studied using a Phenom ProX scanning electron microscope at a 10 kV accelerating voltage. 

### 2.10. Fourier-Transform Infrared Spectroscopy

The infrared (FTIR) spectra of the films were recorded in the 4000–650 cm^−1^ range with a resolution of 2 cm^−1^ using a Spectrum Two spectrometer (Perkin Elmer). For measurements, films were cut into small discs and placed tightly between the sensor and support to ensure good contact. For each spectrum, 25 scans were taken. These analyses were performed in duplicate at room temperature.

## 3. Results and Discussion

**Figure 1:**
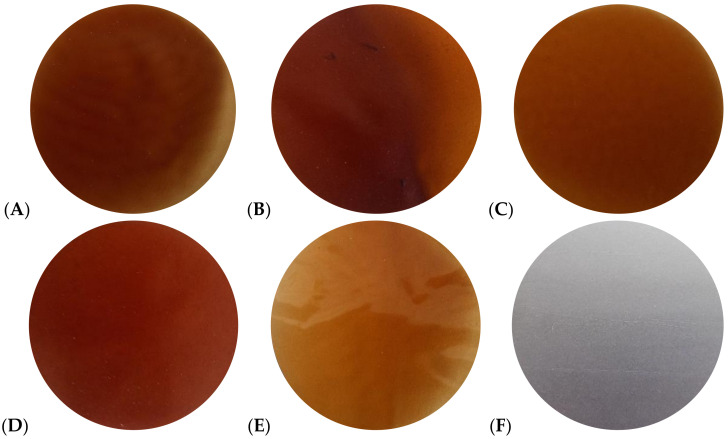
Physical appearance of the modified: (**A**) starch-based (ST) film; (**B**) chitosan-based (CH) film; (**C**) alginate-based (ALG) film; (**D**) starch and chitosan-based (STCH) film; (**E**) starch and alginate-based (STALG) film; (**F**) commercially available PLA-based film.

The images of ST, CH, ALG, STCH, STALG films are shown in Figure 1. As it can be noticed, all obtained films are homogeneous and they have a slightly brownish color due to the addition of a chestnut extract. The thickness of prepared films ranges between 40 µm for alginate films to 110 µm for films based on alginate and starch. The commercially available PLA-based film had a thickness of 7 µm.

### 3.1. Moisture Content, Swelling Degree and Total Soluble Matter

**Figure 2:**
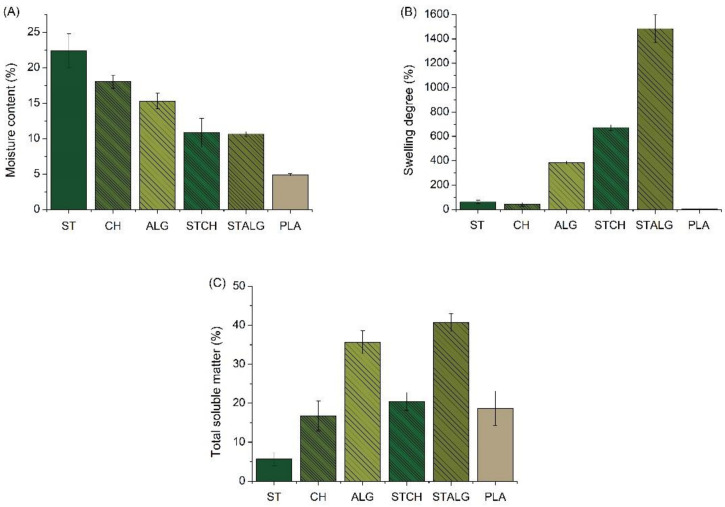
Moisture content (**A**), swelling degree (**B**) and total soluble matter (**C**) of the starch-based (ST), chitosan-based (CH), alginate-based (ALG), starch and chitosan-based (STCH) and starch and alginate-based (STALG) films and the commercially available PLA-based film (*p* < 0.05).

The MC, SD and TSM values of the obtained films are shown in Figure 2. These material properties are related to the film’s sensitivity to water and moisture, which is one of the major areas of interest in the design of food packaging materials. MC indicates the total volume of empty space occupied by water molecules in the network microstructure of the film [50]. As shown in Figure 2A, the MC values are the lowest for the commercially available PLA-based sample (about 5%) and the highest for the starch-based sample (about 22%). The chitosan and alginate-based samples also showed higher moisture content than PLA (about 17% and 16%, respectively). It is worth noting that the samples based on two polysaccharides, i.e., STCH and STALG, showed much lower MC values than these polymers separately (about 10% for both samples). Similar MC values were observed for other films based on starch [51], chitosan [52,53], alginate [54,55] and their blends [56,57]. In the case of SD (Figure 2B), which is an undesirable property of a film, especially if it is intended for packaging applications [58], it can be noticed that the highest value of this parameter is obtained for STALG and STCH (about 1400% and 700%, respectively). Additionally, in this case, the different behavior of the two-component samples compared to the single-polymer based samples can be observed. Samples based on a single polysaccharide had a much lower SD value than their combinations. The lowest SD value was observed for PLA (i.e., about 5%), and the nearest values were observed for ST and CH (about 60% and 40%, respectively).

Balakrishnan et al. [59] also prepared films using starch as a matrix and oxidized sucrose as a crosslinking agent. After crosslinking, a reduction in film swelling was observed, indicating that the acetal bond had a strong influence on the swelling behavior of the films, acting as a barrier that prevents water molecules from passing through, causing the films to swell [19,48,59]. In this case, swelling was reduced by up to half for films prepared with oxidized sucrose. On the other hand, Rodríguez et al. [60] studied the SD of chitosan films modified with different content of glycerol and observed that the amount of glycerol significantly affected the swelling behavior of the film. Li et al. [61] studied the influence of the crosslinking agent content (calcium chloride) on the swelling behavior of alginate-based films. They noticed that the SD values of the films decreased significantly when the concentration of CaCl_2_ was increased to 6% *w*/*v*. The results indicated that the total degree of cross-linking of the films increased with CaCl_2_ concentration. 

TSM is another important factor in selecting films for specific applications. Solubility is a desirable property in many cases, such as for food encapsulation. However, for foods with high moisture content, water resistance and integrity are required, and high solubility of the film is disadvantageous. In our case, low solubility in water is desirable, and considering the TSM values (Figure 2C), it can be noticed that this parameter is similar for CH, STCH and PLA samples (about 18%). However, the lowest value was observed for ST film (about 5%) and the highest value for ALG and STALG (about 35 and 40%, respectively). These values show that each modification had the desired effect, as previous studies indicate that the films based on the pure polysaccharides used in this study are almost completely soluble [62]. ST and CH samples show the lowest SD and TSM values among all obtained samples, indicating their best properties for use as food films. Compared to the commercially available PLA sample, the TSM properties of the ST film are even better (Figure 2C), indicating that the films obtained in the present study fully meet the hydrophobicity requirements of commercially available films.

### 3.2. Contact Angle 

**Figure 3:**
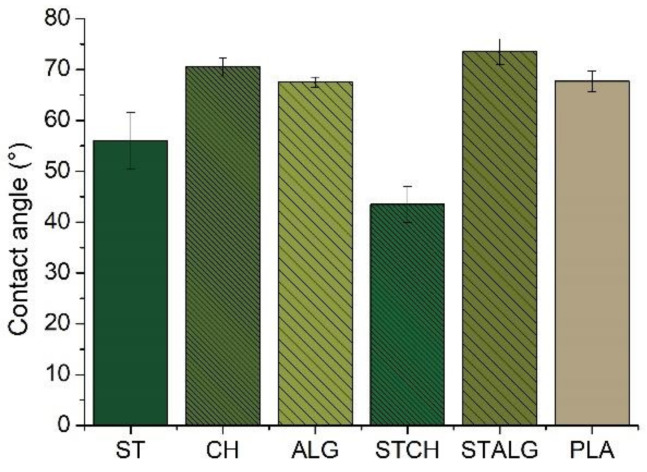
Contact angle of the starch-based (ST), chitosan-based (CH), alginate-based (ALG), starch and chitosan-based (STCH) and starch and alginate-based (STALG) films and the commercially available PLA-based film (*p* < 0.05).

The water contact angle values of the obtained films are shown in Figure 3 to understand the effect of the modifiers on the wettability of the films. This parameter is a good indicator of the hydrophilicity, because as the wettability of the surface increases, the contact angle decreases [53]. It is well known that the water contact angle (θ) value of θ < 90° indicates the hydrophilic nature of the test sample, while θ > 90° represents its hydrophobic nature [63,64]. The values for all investigated films were less than 90°, indicating hydrophilicity of the surfaces. The most hydrophilic film was found to be STCH and ST, with a contact angle of about 40° and 55°, respectively. Bastos et al. [65] observed that the unmodified starch film had a contact angle of 55°, which decreased rapidly with time if the droplet remained on the surface due to water absorption. The addition of glycerol increased the hydrophilic behavior of the film [66,67], but in our case the additional modification with oxidized sucrose caused the contact angle value to remain the same as the unmodified film. On the other hand, Balakrishnan et al. [59], who examined the contact angle of films based on pure starch and modified with oxidized sucrose, showed that the reduction in hydroxyl groups in the crosslinked sample due to the reaction between starch and oxidized sucrose resulted in a reduction in hydrophilic properties. The pure starch film without modifier showed the lowest contact angle (about 33°), which was due to the presence of a large number of hydroxyl groups in starch. For films prepared with oxidized sucrose, the contact angle increased to about 92°. The authors hypothesized that [59] that during film drying the aldehyde groups in the oxidized sucrose sample reacted with the hydroxyl groups of starch and formed water stable acetal bonds. Other samples, including PLA, shows a contact angle of approximately 70°. Although this value still indicates the hydrophilic nature of the film, the use of PLA films in the packaging market indicates that this level of hydrophilicity is sufficient for food packaging. Therefore, it can be concluded that the CH, ALG and STALG films obtained in this study should be the best alternative to commercially available films, including those based on PLA. Previous studies [53,68,69] demonstrated that the contact angle for pure chitosan film was approximately 105°. As estimated in our research, the contact angle values for chitosan equals to 71° due to the modification with hydrophilic glycerol. Taverna et al. [70] determined the contact angle for films based on pure alginate, which was about 25°. Its value increased after treatment of the film with CaCl_2_, which is related to the lower water absorption capacity of the crosslinked films. Such a relationship can also be observed in our study, in which the contact angle for ALG and STALG was about 70°. 

### 3.3. Oxygen and Carbon Dioxide Permeability

**Figure 4:**
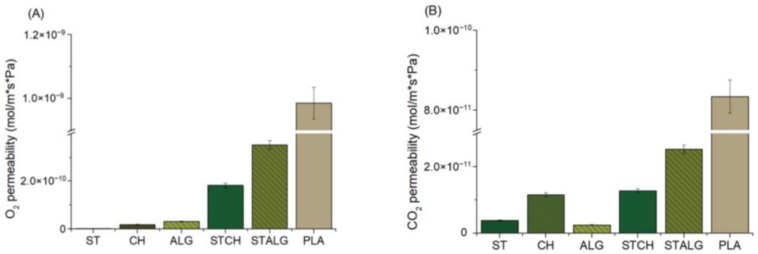
Oxygen (**A**) and carbon dioxide (**B**) permeability of the starch-based (ST), chitosan-based (CH), alginate-based (ALG), starch and chitosan-based (STCH) and starch and alginate-based (STALG) films and the commercially available PLA-based film (*p* < 0.05).

Oxygen and carbon dioxide permeability values are shown in Figure 4. Oxygen and carbon dioxide are the most common gases studied in food packaging applications. The permeation of oxygen from the air into the package should be avoided due to deterioration of product properties caused by oxidation during long-term storage. On the other hand, carbon dioxide can potentially reduce the food degradation, leading to a significant increase in shelf life [71,72]. Maintaining a certain level of carbon dioxide concentration along with the desired oxygen concentration that have been introduced during food packaging is helpful in preserving different types of food. Therefore, high barrier properties of the film are very important for food packaging films [73]. The highest oxygen and carbon dioxide permeabilities were observed for PLA, indicating its low barrier properties compared to the films obtained in the present study. All other films provided good barrier properties to O_2_ and CO_2_. ST films showed almost no detectable O_2_ permeability, while CH and ALG films showed permeability values of about 2 and 3 × 10^−10^ cm^3^ /m⋅s⋅Pa, respectively. The CO_2_ permeability was much lower than that of O_2_, i.e., about 1 × 10^−11^ cm^3^ /m⋅s⋅Pa and 2.5 × 10^−12^ cm^3^ /m⋅s⋅Pa, respectively. This effect was also observed for other similar polymers previously [72,74]. 

O_2_ permeation is generally lower when compared to CO_2_ permeation, due to the decrease in diffusivity and increase in solubility with decreasing permeant size (O_2_ molecular diameter is 3.1 Å and CO_2_ molecular diameter is 3.4 Å, respectively) [75]. García et al. [76] used plasticizers (glycerol and sorbitol) to improve the barrier properties of starch-based films. They observed that O_2_ permeability was significantly lower (10^−10^ cm^3^ /m⋅s⋅Pa) than for CO_2_ (10^−9^ cm^3^ /m⋅s⋅Pa), indicating a selective effect of these films on gas permeability, which the authors attributed to the higher solubility of CO_2_ in the starch films. It was also observed that the CO_2_ and O_2_ permeabilities for the unplasticized films were significantly higher than those for the plasticized films, which was attributed to the presence of pores and cracks on the surface of the unplasticized films. On the other hand, Butnaru et al. [77] investigated the effect of plasticizer for O_2_ and CO_2_ permeability of chitosan films. The O_2_ permeability values for the reference chitosan film were 67 mL/m^2^, and after the addition of plasticizer, the O_2_ permeability was higher at 212 and 134 mL/m^2^, respectively. The increased O_2_ values can be attributed to the plasticization effect of the biopolymer by the plasticizer molecules [78]. The plasticization effect results in increased mobility of oxygen molecules due to disruption of hydrogen bonds, creating additional sites for oxygen solvation [79]. In turn, the presence of the plasticizer led to improved barrier properties for CO_2_, achieving half the CO_2_ values compared to pure chitosan [77]. It is worth noting that the films based on two polymers showed a different characteristics than the films based on a single polymer. In the case of oxygen permeability for films based on two polysaccharides (Figure 4A), the barrier properties of STCH and STALG films decreased significantly, by as much as about 70% and 90%, respectively, compared to ST, CH and ALG films. On the other hand, the lowest permeability values for carbon dioxide (Figure 4B) were recorded for ST and ALG, but the combination of these two polymers (STALG) resulted in a significant increase in permeability. In contrast, the high barrier properties of starch in the ST sample did not contribute to the reduced permeability of carbon dioxide when combined with chitosan. STCH sample showed barrier properties at the same level as CH. These results could mean a lower degree of interaction between starch and chitosan than in the case of starch and alginate, causing easier migration of oxygen and carbon dioxide molecules through the film. Nevertheless, the results obtained for all the investigated films in the present study outperformed the commercial food packaging films used today, such as PLA films or even low-density polyethylene (LDPE) [53,80]. 

### 3.4. Mechanical Properties

**Figure 5:**
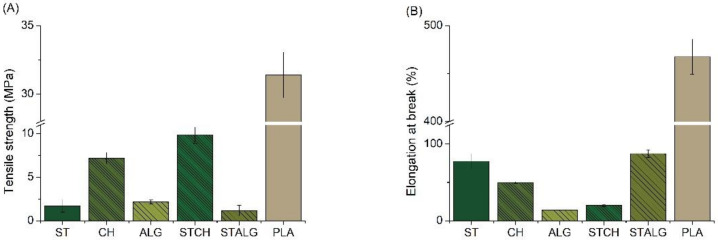
Tensile strength (**A**) and elongation at break (**B**) of the starch-based (ST), chitosan-based (CH), alginate-based (ALG), starch and chitosan-based (STCH) and starch and alginate-based (STALG) films and the commercially available PLA-based film (*p* < 0.05).

Mechanical properties of investigated films are summarized in Figure 5. They are important in evaluating the quality of food packaging films, since they are used to assess the ability of packaging materials to maintain integrity under various stresses that occur during processing and storage of packaged foods [77]. It can be seen that the tensile strength values (Figure 5A) are the highest for the commercially available PLA-based film (about 30 MPa). This value is about 70% higher when compared with CH and STCH and as much as 95% higher when compared with ST, ALG and STALG. The lowest tensile strength values were noted for ST and ALG (about 2 MPa) and the highest for CH (about 8 MPa). The combination of the weakest samples, i.e., ST and ALG, did not improve their properties, since the tensile strength of STALG remained around 5 MPa. On the other hand, the combination of ST and CH slightly improved the tensile strength to 12 MPa (as noted for STCH). This shows that in some cases the addition of a polymer with a weaker tensile strength can increase the mechanical properties of the final material. The same relationship was observed by Tan et al. [81], who investigated the effects of starch concentration, chitosan and glycerol content on mechanical properties of their composites. An optimum tensile strength of 5.19 MPa was obtained with the combination of these two polysaccharides, in which the concentrations were: starch 5 wt.%; chitosan 20 wt.%; glycerol 40 wt.%. Elongation at break results (Figure 5B) also show that the highest values were noted for the commercial PLA sample (about 460%). In this case, the value is also significantly higher than for the samples obtained in this study (by about 80% when compared to ST, CH, STALG and by as much as about 95% for ALG and STCH). Djalila et al. [37] studied the physicomechanical properties of films based on starch, alginate and their mixture, and noted a clear increase in the elongation at break from about 5% to 19% of the films when the alginate content increased from 0% to 50%. On the other hand, Xu et al. [82] investigated and compared the mechanical properties of chitosan and starch-based films at different ratios and at a concentration gradient of 0.5. The results showed that the tensile strength and elongation at break of the films first increased and then decreased with increasing starch content. The maximum elongation at break of the film with a starch to chitosan weight ratio of 1.5:1 reached 60%. Balakrishnan et al. [59] studied the effect of crosslinking starch with oxidized sucrose on the mechanical properties of investigated films and found a significant improvement after adding the modifier to pure starch. A maximum threefold increase in tensile strength was obtained with a slight deterioration in elongation at break. 

The increase in strength properties is mainly due to the good compatibility of starch with oxidized sucrose, which results from chemical and physical interactions between starch and the crosslinking agent. On the other hand, crosslinked films have a slightly lower elongation percentage due to the limited mobility of the starch macromolecular chain. Rhim [83] studied alginate films prepared with and without CaCl_2_ treatment and demonstrated an increase in the tensile strength of and a concurrent decrease in elongation at break when CaCl_2_ was applied to the alginate films. The same observation was also found in our study, in which the elongation at break also decreases with an increase in tensile strength. Another property was observed by Ibrahim et al. [33], who also studied alginate films modified with CaCl_2_. In this case, the alginate films showed an increase in tensile strength with an increase in elongation at break after modification. The mechanical properties of the alginate films increased up to 4 times after immersion in CaCl_2_. The improvement in the mechanical properties of the films may be due to the crosslinking reaction between Ca^2+^ ions and the carboxyl group of alginate. The crosslinking can be formed by simple ionic bridging of two carboxyl groups on adjacent polymer chains with calcium ions [84]. Even though polysaccharide-based films have not been found to outperform commercial PLA films from the point of view of their mechanical properties, acquired results did not stand out of other literature proceedings describing this class of materials. The best tensile strength results were obtained for CH and STCH (i.e., about 8 MPa and 10 MPa, respectively), and the best elongation at break properties were obtained for ST and STALG (i.e., about 78% and 88%, respectively). Obviously, further research is needed to improve their properties, but the results obtained so far can be considered as satisfying.

### 3.5. Antimicrobial Properties

**Figure 6:**
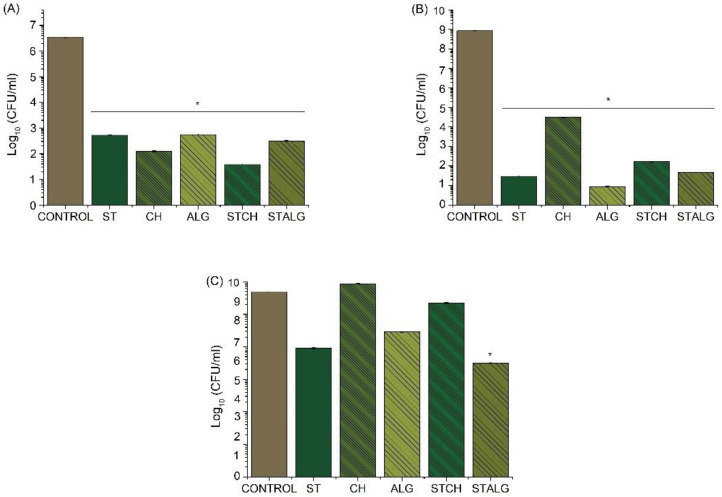
The effect of modified polysaccharide films on the viability of *S. epidermidis* cells (**A**), *E. coli* cells (**B**) and *C. albicans* (**C**); unpaired *t*-Test: * *p* < 0.05.

The results of antimicrobial assays demonstrated that different films obtained in this study caused a significant reduction in the growth of both model Gram-positive and Gram-negative bacteria, such as *S. epidermidis* (Figure 6A), and *E. coli* (Figure 6B). In particular, a 4–5 log reduction in the growth of *S. epidermidis* was observed while for *E. coli* there was a 5 log reduction for CH and over a 7–8 log reduction when co-cultured with starch-based, alginate-based, and both polymer blend films. Moreover, the study showed that starch-based films were more resistant to *E. coli* bacterial pathogens than chitosan-based films. 

It is important to indicate that the primary source of antibacterial activity of investigated films was the presence of a chestnut extract, which is a rich source of polyphenolic compounds, phenolic acids, and tannins [85,86]. Since the amount of chestnut extract was the same in all investigated films, the difference in antibacterial effects should be associated with a biopolymer matrix and the presence of modifiers. For instance, Priya et.al. [87] studied the antimicrobial activity of PVA starch cross-linked samples and found that both the crosslinking agent and plasticizer induced antimicrobial properties. In our study, starch was crosslinked with oxidized sucrose, which led to acetal formation able to release cationic ions interacting with the anionic charges of microbial cell membranes through electrostatic bonding. This specific interaction led to increased cell peripherality and leakage of intercellular components, which ultimately induced cell death [88]. It is also expected that the low film permeability could reduce the attraction of bacterial species [89]. 

On the other hand, the antibacterial properties of chitosan could be easily justified by its ability to bind to the negatively charged bacterial cell wall and cause disruption of the cell, followed by its interactions with DNA leading to cell death [90]. It is also known that the presence of chitosan can affect an efflux of particular ions (K^+^, Ca^2+^, H^+^, Cl^-^) and increase transmembrane potential difference in the cells, altering the ionic balance required for microorganisms to maintain their vitality [91]. Still, the antibacterial properties of chitosan are dependent on its molecular weight, degree of deacetylation, concentration, pH, chitosan source, temperature, as well as a type of microorganisms and a cell growth phase [92]. Superior antibacterial properties of ALG foils when compared with other investigated materials could be associated with the presence of CaCl_2_. Recent studies [93,94] show that CaCl_2_ exhibits an inhibitory effect on microbial growth, as demonstrated in the example of *Ralstonia pseudosolanacearum.* The antibacterial activity mechanism of CaCl_2_ is based on its ability to arrest the swarming motility of bacteria, disrupt their physiological functions and, finally, inhibit the formation of a mature biofilm. 

Although a chestnut extract was found to exhibit antifungal properties, not all investigated polysaccharide-based foils were observed to follow this behavior (Figure 6C). An antifungal effect was noted for STALG, ST and ALG, but no effect of antifungal activity was observed in any of chitosan-based foils, even though chitosan itself is known to exhibit antifungal properties [91]. We expect this behavior to be associated with a limited solubility of chitosan at neutral pH, which restricts its intrinsic antifungal activity and limits the release of a chestnut extract.

The results of microbiological investigations confirmed that fabricated polysaccharide-based films can be regarded as having antibacterial activity towards *E. coli* and *S. epidermidis*. It should be highlighted that commercially used packaging materials, for instance, PLA, alone and unmodified did not show antimicrobial activity [95]. Therefore, modified polysaccharide-based films, as confirmed by previous studies (Table 2), perfectly match current trends in packaging industry to apply bioactive and biodegradable materials for the development of antibacterial and antioxidative packaging systems functionalized with natural bioactive agents. 

### 3.6. Scanning Electron Microscopy

**Figure 7:**
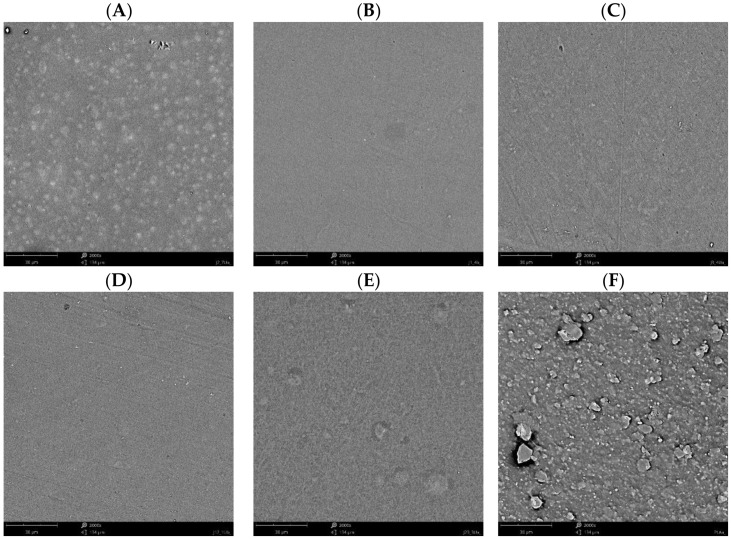
SEM images of: (**A**) starch-based (ST) film; (**B**) chitosan-based (CH) film; (**C**) alginate-based (ALG) film; (**D**) starch and chitosan-based (STCH) film; (**E**) starch and alginate-based (STALG) film; (**F**) commercially available PLA-based film.

Figure 7 shows scanning electron microscopy (SEM) images of the obtained polysaccharide-based and commercially available PLA films. For all the obtained samples, a homogeneous structure is observed, indicating that the modified films have a smooth, flat surface in the case of the ST film, the presence of undissolved particles of chestnut extract in the polymer matrix is noted. Nevertheless, the blends of starch with other polymers, i.e., chitosan or alginate (sample STCH and STALG, respectively) show that the extract appears to be completely dissolved. For the commercially available PLA film, unspecified particles are noted which may indicate the use of fillers in the present film to modify the physicochemical properties of the material. As noted, when analyzing mechanical properties of samples, PLA film was found to have the highest mechanical properties, which is most likely due to the presence of a filler. Many studies [101,102,103] noted that fillers significantly improve the mechanical properties of samples, including materials based on polysaccharides. Nevertheless, the SEM images confirm the homogeneity of the chestnut extract dispersion in the polymer matrix and the compatibility of the polymers. 

### 3.7. Fourier-Transform Infrared Spectroscopy

**Figure 8:**
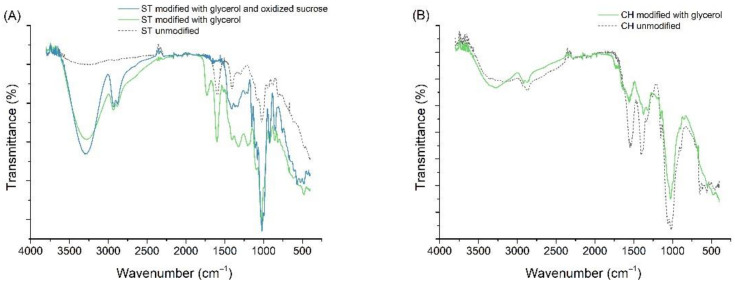

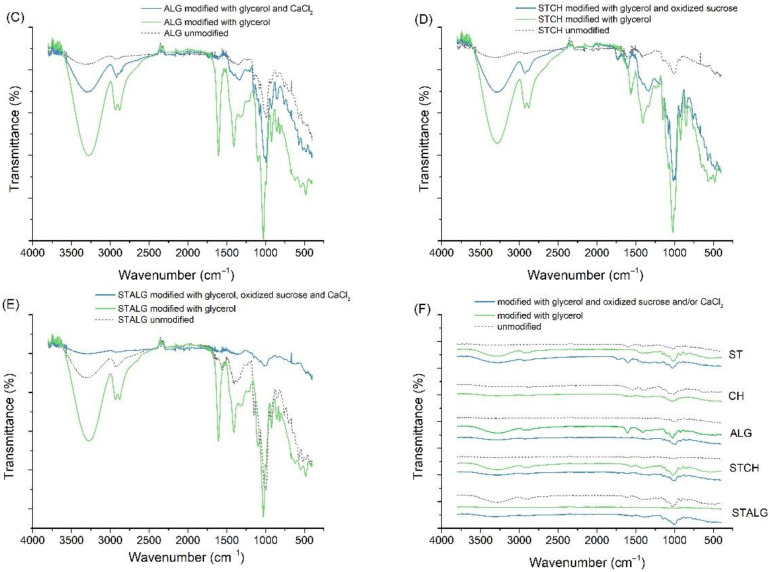
FTIR spectra of ST films (**A**), CH films (**B**), ALG films (**C**), STCH films (**D**), STALG films (**E**) and a summary of all spectra (**F**).

FTIR spectra are useful for observing and understanding the molecular interactions that polymers engage in with various modifications. Figure 8 illustrates the FTIR spectra of unmodified and modified polysaccharide films and their blends, as well as a comparison of all obtained samples. The FTIR spectra of unmodified and modified starch films (Figure 8A) showed clear changes in the spectral regions primarily due to the use of oxidized sucrose and glycerol, as a peak at 3450 cm^−1^ is noted, which is attributed to the stretching vibration of the -OH groups, and at 2960 cm^−1^, which can be attributed to the stretching vibration of the -CH bond [18,104]. The absorption band between 1000 and 1200 cm^−1^ was characteristic of -CO stretching of the polysaccharide skeleton [105]. 

FTIR spectra of unmodified and modified chitosan films (Figure 8B) are almost comparable, indicating that glycerol did not change the structure of chitosan but only interacted with it. Chitosan exhibited two strong vibrational bands at 1645 and 1584 cm^−1^; these bands are attributed to the vibrations of amide I and amide II, respectively [106]. It has been reported that the deformation vibrations of amines usually produce strong or very strong bands in the region of 1638–1575 cm^−1^ [107]. A broad band in the range of about 3600–3100 cm^−1^ is attributed to N-H and OH-O stretching vibrations. This band is also related to some extent to the intermolecular hydrogen bonds of the chitosan molecules [108]. 

The spectra of alginate films are shown in Figure 8C. As alginate is modified with glycerol, the O-H stretching peak (~3380 cm^−1^) has a much higher intensity due to the O-H groups derived from glycerol. On the other hand, when additionally modified with calcium chloride, the O-H stretching peak becomes narrower and of greater intensity compared to the unmodified sample, but lower than when modified with glycerol by itself. This is characteristic of an increase in intramolecular bonding [109]. The O-H shoulder (at 3250 cm^−1^), corresponding to the intermolecular binding, also becomes narrower and has higher intensity. The -COO^−^ peaks at ~1650 and 1420 cm^−1^ (asymmetric and symmetric stretching, respectively) become broader and more intense after glycerol and their intensity strongly decreases after additional modification with calcium chloride. This is because this peak is characteristic of the ionic bonding. When calcium ions replace sodium ions in the alginate structure, the charge density, radius and atomic mass of the cation change, creating a new environment around the carbonyl group [109]. In the region 1150–1000 cm^−1^, a set of intense peaks can be observed that can be attributed to C-C and C-O stretching vibrations [110].

The FTIR spectra obtained for STCH are shown in Figure 8D. It can be observed that the unmodified STCH film as well as the modified film show a broad absorption band centered at 3380 cm^−1^, which can be attributed to N-H and O-H stretching vibrations [111]. The peak is definitely more intense when modified with glycerol alone. After modification with oxidized sucrose, the intensity decreases suggesting higher interactions with O-H groups for the STCH sample than for the ST sample (Figure 8A). The peak at 2918 cm^−1^ can be attributed to the asymmetric C-H stretching vibrations of methylene groups, and the peak at about 2876 cm^−1^ to the -CH_3_ group of acetylamino groups in chitosan [18,104] Their amount decreased in the modified film with glycerol and oxidized sucrose compared to the modified only with glycerol sample, which may be due to the reaction of chitosan with sodium periodate, which leads to the elimination of acetamido groups by strong oxidant and strong acid [112]. The band at 1645 cm^−1^ can be attributed to the C-O stretching vibration of the residual amide bond [113]. On the other hand, the band at 1597 cm^−1^ can be attributed to N-H bending vibrations of chitosan, which disappeared in the modified sample with oxidized sucrose by reaction with aldehydes from oxidized sucrose products [112]. The band at 1412 cm^−1^ is a C-N stretching vibration in chitosan and the peak at 1080 cm^−1^ is attributed to C-O stretching vibration [113]. The bands in the 1000 and 1100 cm^−1^ region are attributed to the C-O bond of the C-O-H group in starch [114]. The intensity of these bands is significantly reduced for the unmodified sample, suggesting high interactions between the starch itself with chitosan.

The spectra of modified and unmodified starch- and alginate-based films are shown in Figure 8E. A strong absorption due to -OH stretching vibration (3400 cm^−1^) was noted in the FTIR spectra. It is typical of polymeric association of hydroxyl groups [115]. As can be seen, the highest intensity was recorded for the sample modified with glycerol only, and the additional modification with calcium chloride caused the intensity to drop below that recorded for the unmodified sample. This explains the hydrophilicity results of these films, which were found to be lower than for the STCH sample. The peaks at values from about 1600 cm^−1^ to 1400 cm^−1^ can be attributed to asymmetric and symmetric stretching vibrations of -COO^−^ [116]. The spectra of the modified blend with glycerol, oxidized sucrose and calcium chloride exhibit lower intensities, which indicates intermolecular interaction through hydrogen bond formation between -COO^-^ groups in alginate and -OH groups in starch [115].

## 4. Conclusions

In this study, modified films based on starch, chitosan, alginate and their compositions with a chestnut extract were prepared by a solution casting method. An extensive characterization of as-formed films revealed that polysaccharides are promising materials for food packaging industry. Particular materials were shown to match the requirements of commercially available films, for example low swelling degree (starch and chitosan), low total soluble material (starch), optimal hydrophilicity (chitosan alginate, and starch/alginate), and high barrier properties with respect to O_2_ and CO_2_ (all investigated films). An important benefit of polysaccharide-based films containing chestnut extract was their antibacterial nature verified against *E. coli*, *S. epidermidis*, and *C. albicans.* Even though the mechanical properties of investigated materials were satisfactory, they were still worse than the ones of a commercially available PLA film. However, SEM studies showed that the commercially available PLA film contains particles indicating the use of a filler, which can significantly improve the mechanical properties. Therefore, further studies with the use of fillers are planned for starch, chitosan, alginate films and their blends.

Due to the fact that polysaccharides are becoming an increasingly popular alternative to traditional plastics in packaging production, the research presented herein may contribute to expanding the applicability of biopolymers through an easier selection of the polymer matrix. This is a comparative study and further research, especially in direction of improving mechanical properties, is needed for further application of polysaccharides in industry.

## Figures and Tables

**Table 1:** Abbreviations of samples investigated in this study.

Abbreviations	ST	CH	ALG	STCH	STALG
Sample	Modified starch-based film	Modified chitosan-based film	Modified alginate-based film	Modified starch and chitosan-based film	Modified starch and alginate-based film

**Table 2:** Polysaccharide-based films containing different plant extracts exhibiting antimicrobial activity towards particular microorganisms.

Biopolymer	Antimicrobial Agent	Microorganisms	Ref.
Chitosan	Chestnut extract	*E. coli, B. subtilis*	[96]
Chitosan	Chestnut extract	*P. fluorescens, E. coli, P. commune*	[97]
Chitosan	Chestnut extract	*E. coli, S. epidermidis*	This study
Chestnut starch-chitosan	*Cornus officinalis* fruit extract, glycerol monolaurate, nisin, pine needle essential oil	*E. coli* *, S. aureus, L. monocytogenes*	[98]
Starch	Chestnut extract	*E. coli, S. epidermidis*	This study
Alginate	Nisin, ε-polylysine, olive extract, nettle extract, green tea extract	*E. coli, S. aureus*	[99]
Alginate	Algae extract, aminoglycosides	*E. coli*	[100]
Alginate	Chestnut extract, CaCl_2_	*E. coli, S. epidermidis, C. albicans*	This study

## Data Availability

Not applicable.

## Supplementary Materials

The following supporting information can be downloaded at: www.mdpi.com/article/10.3390/ma15093236/s1, Figure S1: Schematic diagram of experimental apparatus for gas permeability testing; Figure S2: Antimicrobial activity of four commercially available extracts: chestnut (K), graviola (G), grape (W), nettle (P) tested against model Gram-positive bacteria (*S. epidermidis*), Gram-negative bacteria (*E. coli*) and yeasts (*C. albicans*); control well was filled with deionized water (ddH_2_O).
