# Supplementary material for: Antibacterial and Biodegradable Polysaccharide-Based Films for Food Packaging Applications: Comparative Study
Source: Materials (Basel). 2022 Apr 29;15(9):3236. doi: 10.3390/ma15093236 (PMC9103775; doi:10.3390/ma15093236)
Supplement: Supplementary file 1 [file materials-15-03236-s001.zip › materials-1690517-supplementary.pdf]

SUPPLEMENTARY INFORMATION

Antibacterial and Biodegradable Polysaccharide-Based Films for Food Packaging Applications: Comparative Study

Weronika Janik, Michał Nowotarski, Divine Yutefar Shyntum, Angelika Banaś, Katarzyna Krukiewicz, Stanisław Kudła, Gabriela Dudek

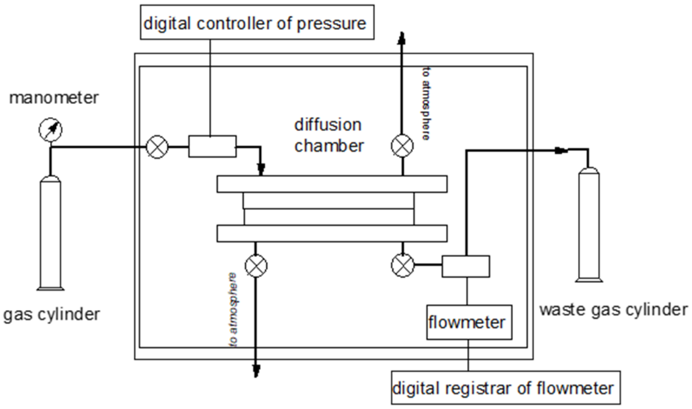

Figure S1. Schematic diagram of experimental apparatus for gas permeability testing.

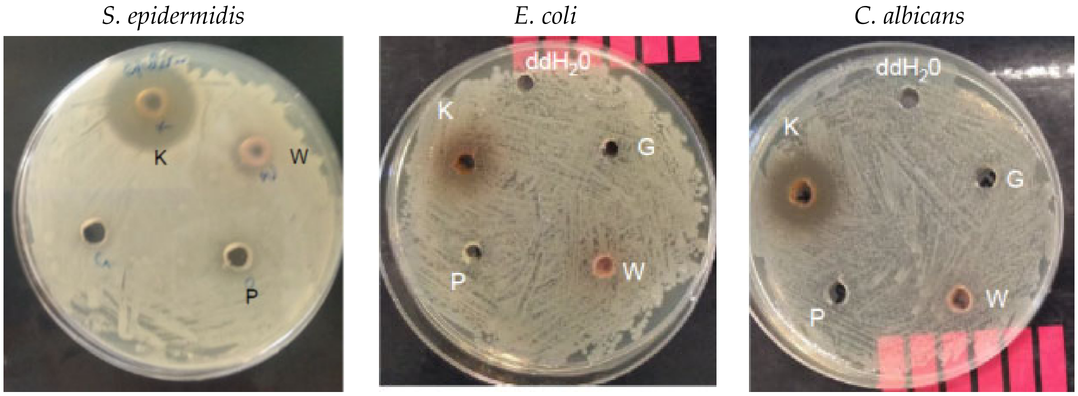

Figure S2. Antimicrobial activity of four commercially available extracts: chestnut (K), graviola (G), grape (W), nettle (P) tested against model Gram-positive bacteria (*S. epidermidis*), Gram-negative bacteria (*E. coli*) and yeasts (*C. albicans*); control well was filled with deionized water (ddH$_2$O).
